# Combining measurements from three anatomical areas for glaucoma diagnosis using Fourier-domain optical coherence tomography

**DOI:** 10.1136/bjophthalmol-2014-305907

**Published:** 2015-03-20

**Authors:** Nils A Loewen, Xinbo Zhang, Ou Tan, Brian A Francis, David S Greenfield, Joel S Schuman, Rohit Varma, David Huang, for the Advanced Imaging for Glaucoma Study Group

**Affiliations:** 1 Department of Ophthalmology, UPMC Eye Center, Ophthalmology and Visual Science Research Center, University of Pittsburgh School of Medicine, Pittsburgh, Pennsylvania, USA; 2 Casey Eye Institute, Oregon Health & Science University, Portland, Oregon, USA; 3 Doheny Eye Institute, University of Southern California, Los Angeles, California, USA; 4 Doheny Eye Institute, University of California, Los Angeles, California, USA; 5 Department of Ophthalmology, Bascom Palmer Eye Institute, University of MiamiMiller School of Medicine, Palm Beach Gardens, Florida, USA; 6 USC Eye Institute and the Department of Ophthalmology, Keck School of Medicine, University of Southern California, Los Angeles, California, USA

## Abstract

**Aims:**

To improve the diagnostic power for glaucoma by combining measurements of peripapillary nerve fibre layer (NFL), macular ganglion cell complex (GCC) and disc variables obtained with Fourier-domain optical coherence tomography (FD-OCT) into the glaucoma structural diagnostic index (GSDI).

**Methods:**

In this observational, cross-sectional study of subjects from the Advanced Imaging of Glaucoma Study, GCC and NFL of healthy and perimetrical glaucoma subjects from four major academic referral centres of the Advanced Imaging of Glaucoma Study were mapped with the RTVue FD-OCT. Global loss volume and focal loss volume parameters were defined using NFL and GCC normative reference maps. Optimal weights for NFL, GCC and disc variables were combined using multivariate logistic regression to build the GSDI. Glaucoma severity was classified using the Enhanced Glaucoma Staging System (GSS2). Diagnostic accuracy was assessed by sensitivity, specificity and the area under the receiver operator characteristic curve (AUC).

**Results:**

We analysed 118 normal eyes of 60 subjects, 236 matched eyes of 166 subjects with perimetrical glaucoma, and 105 eyes from a healthy reference group of 61 subjects. The GSDI included composite overall thickness and focal loss volume with weighted NFL and GCC components, as well as the vertical cup-to-disc ratio. The AUC of 0.922 from leave-one-out cross validation was better than the best component variable alone (p=0.047). The partial AUC in the high specificity region was also better (p=0.01), with a sensitivity of 69% at 99% specificity, and a sensitivity of 80.3% at 95% specificity. For GSS2 stages 3–5 the sensitivity was 98% at 99% specificity, and 100% at 95% specificity.

**Conclusions:**

Combining structural measurements of GCC, NFL and disc variables from FD-OCT created a GSDI that improved the accuracy for glaucoma diagnosis.

## Introduction

It has been estimated that about half of patients with glaucoma in the USA do not know that they have the disease.^1^ To address this public health problem, further improvement in objective diagnostic technology is needed. The accuracies of quantitative imaging for glaucoma detection may often still not be sufficient for initial diagnosis without further testing.^2,3^ Although structural optical coherence tomography (OCT) tends to outperform other testing, its diagnostic sensitivities were found to be only 57–83%^4,5^ at a fixed specificity of 95% for detecting perimetrical glaucoma (PG). Ganglion cell complex (GCC) analysis has been found to perform similarly to nerve fibre layer (NFL) in several studies.^6–8^ At the 99% and 95% specificity cut-offs, Fourier-domain OCT (FD-OCT) diagnostic sensitivity of single OCT structural variables were still relatively low with 0.13 and 0.34, respectively,^8^ but because of the higher acquisition speeds of 26 000 scans per second, this technology allows to assess different anatomical structures affected by glaucoma rapidly in the same session.

In this investigation, we sought to improve FD-OCT diagnostic accuracy by combining structural data from three anatomical regions affected by glaucoma: (1) the macular GCC layer that contains the retinal ganglion cell bodies, (2) the NFL consisting of the ganglion cell axons and (3) the cup and disc.

## Methods

### Subjects

In this observational, cross-sectional study, Advanced Imaging for Glaucoma Study (AIG Study) baseline visit data that was obtained from AIG Study participants from the University of Southern California, University of Miami and University of Pittsburgh Medical Center, who had been recruited between 2003 and 2012 at the time of the FD-OCT scan, were analysed. The design and baseline participant characteristics were described recently.^9^ A subset (N) of subjects was randomly selected from the AIG Study normal group and frequency-matched to the PG group by demographic characteristics for the purpose of testing diagnostic accuracy. A separate reference (R) subset was also randomly selected from the AIG Study normal group (N) and used to define the global loss volume (GLV) and focal loss volume (FLV) variables. The R group was also used to standardise the measured FD-OCT variables.

Inclusion criteria for these groups are described in the AIG Study Manual of Procedures (http://www.aigstudy.net/index.php?id=12, accessed 21 February 2014).^8^ Briefly, subjects in the normal group had an intraocular pressure of less than 21 mm Hg in each eye, a normal standard SITA V.24-2 Humphrey visual field with mean deviation (MD) and pattern SD (PSD) within 95% of the normal reference and a glaucoma hemifield test within 97% limits, respectively. All had a central corneal thickness above 500 μm, a normal-appearing optic nerve head, a normal NFL, an open anterior chamber angle as observed by gonioscopy, and no history of chronic ocular or systemic corticosteroid use. Visual fields were classified by stage using the Enhanced Glaucoma Staging System (GSS2)^10^ that mathematically captures six glaucoma stages with defect severity and defect type using two main perimetrical global indices, MD and PSD.

In contrast, subjects in the PG group had in one or both eyes a glaucomatous visual field with a PSD (p<0.05) or a glaucoma hemifield test (p<1%) outside of normal limits in a consistent pattern on two qualifying Humphrey visual fields, and glaucomatous optic nerve head changes on dilated fundus examination defined as diffuse or localised rim thinning, disc haemorrhage, NFL defect or vertical cup-to-disc ratio (VCDR) greater than the fellow eye by >0.2.

Individuals were excluded if best-corrected visual acuity was less than 20/40, age was less than 40 years or above 79 years, and spherical equivalent refractive error was above +3.00 dioptres or less than −7.00 dioptres. Eyes with diabetic retinopathy or other diseases that could cause visual field loss or optic disc abnormalities, previous intraocular surgery other than an uncomplicated cataract and posterior chamber intraocular lens implantation were also excluded.

The research was conducted in accordance with the Declaration of Helsinki. Informed consent was obtained from all participants after discussing the goals of the study and consequences of participation. Each institutional review board approved the research protocol. Data was handled in compliance with the US Health Insurance Portability and Accountability Act.

Three discrete groups of anatomical variables, NFL, GCC and disc measurements, were acquired by FD-OCT (RTVue system by Optovue, Fremont, California, USA).

### Data acquisition and processing

Data was acquired and processed as detailed in online supplement 1. In the GCC thickness maps and NFL thickness profile data, the GLV was used to measure a pattern of diffuse loss, whereas the FLV was used to measure localised loss^8^ and expressed as percentages of normal. The R group served as the reference standard to define these new variables for NFL. The OCT structural variables were combined into a single glaucoma structural diagnostic index (GSDI) in two stages in order to make optimal use of a large number of variables.

## Results

Among the eligible normal subjects from the AIG Study, 61 subjects (105 eyes) were randomly selected to form the R group. The remaining 60 normal subjects (118 eyes) became the N group (table 1). The PG eyes were then randomly selected within blocks from a larger candidate group of 295 PG eyes to form a cohort with a 2:1 case-control ratio to the N group where the two groups were frequency-matched on age, gender and ethnicity. There were 236 eyes of 166 subjects selected in PG. These numbers of subjects here are slightly different from the baseline for other imaging modalities in the AIG Study^9^ because FD-OCT was not available until late 2006, almost 3 years after the start of the AIG Study recruitment in 2004. Eyes in PG had a significantly lower MD and higher PSD, a lower visual field index and a longer axial length and thinner cornea than eyes in N. There was no intraocular pressure difference (table 1).

Online supplementary table S1 illustrates the diagnostic performance of NFL, GCC and the optic disc variables. NFL GLV, NFL overall thickness and inferior thickness performed the best while the superior thickness had the lowest area under the receiver operator characteristic curve (AUC). Similarly, GCC variables GLV, overall thickness and inferior thickness had the highest AUCs while the superior thickness performed the worst. Among disc variables, the vertical cup to disc ratio and rim volume had the best AUCs while the horizontal cup to disc ratio had the lowest.

We used logistic regression to optimise the weights of NFL and GCC variables to overall, superior and inferior anatomical areas as well as GLV versus FLV (table 2). The regional variables were dominated by NFL while GLV and FLV had more even contributions from GCC and FLV. The composite GLV had the highest AUC followed by FLV. The AUCs of the composite variables were slightly better than the single component variables (see online supplementary table S1), with the biggest improvement seen in FLV.

**Figure 1:**
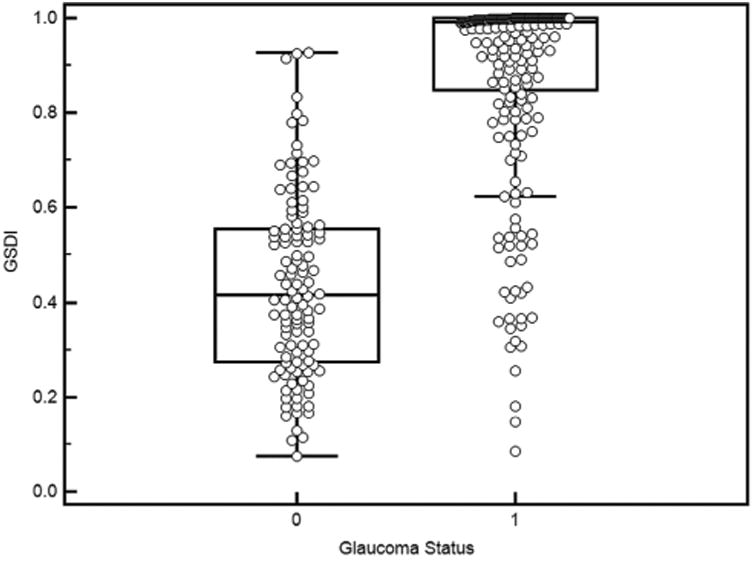
Distribution plot of the glaucoma structural diagnostic index (GSDI). Normal eyes appeared well delineated from glaucoma eyes with a median of 0.155 (25%–75% (0.077, 0.269), Min-Max (0.009–0.815)) compared with a median of 0.978 (25%–75% (0.686, 0.999), Min-Max (0.086–1)).

A multivariate logistic model was used to construct the GSDI. This diagnostic index was calculated from the logistic transformation of a linear combination of a selection of diagnostic variables that optimised the likelihood of correct diagnostic classification. The optimised list of variables included the composite overall thickness, the VCDR, and the composite FLV, in that weight order (table 3). The resulting GSDI formula was: logistic (−0.74*Composite Overall Thickness +0.70×Composite FLV+3.37×VCDR−3.69). The VCDR coefficient in the formula applies to non-standardised VCDR value and therefore differs from the standardised weight in table 3. GSDI had a range from 0 to 1 as shown in the distribution box plot (figure 1) and differentiated normal subjects and PG well. The GSDI values were highly skewed in the PG group, with 75% above the level of 0.8.

**Figure 2:**
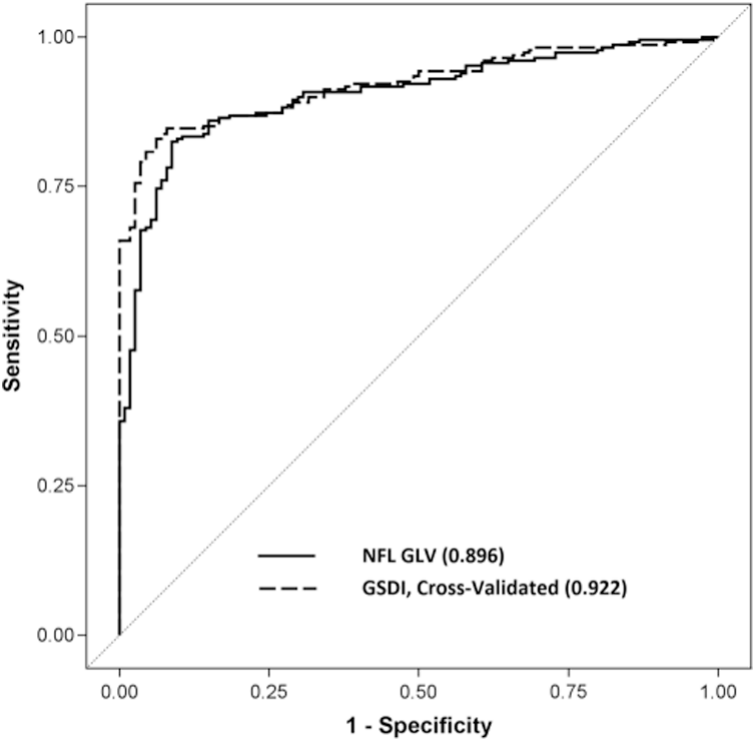
The area under the receiver operator characteristic curve of the of glaucoma structural diagnostic index (GSDI) achieved better sensitivity at higher specificity compared with the nerve fibre layer global loss volume (NFL GLV), the best single diagnostic variable.

The AUC for GSDI was 0.922, significantly better than the AUC for best single component variable variables—NFL GLV (p=0.047). When we graphed the operating characteristics curve for GSDI and compared it with NFL GLV, they were similar in the low specificity region, but GSDI performed noticeably better in the high specificity region (figure 2). The partial AUC (pAUC) for GSDI from 90% to 100% specificity was 0.082 using the cross-validated curve, while the pAUC for the best single parameter NFL_GLV was 0.064. There was a 28% improvement (p=0.013) comparing the pAUC for GSDI over NFL_GLV. The 99% specific cut-off threshold for GSDI was 0.81, above which the eye should be considered abnormal (table 4). The 95% specificity cut-off value for GSDI was 0.59. At 99% specificity, GSDI had 69% diagnostic sensitivity, nearly 17% better than GCC GLV, the best component variable. At 95% specificity, GSDI had 80.2% sensitivity, which was 11% better than NFL GLV.

We further examined the diagnostic performance of the GSDI among PG eyes at different severity stages (table 5) as defined by the GSS2 visual field staging system. Above stage 2, the AUCs were near 0.999, resulting in almost 98% detection of patients with PG at 99% specificity. The Hosmer-Lemeshow goodness-of-fit test,^11,12^ a measure of model fitness for the GSDI multivariate logistic model, demonstrated a p value of 0.58, which suggests that there was no significant evidence to deem the model unfit.

**Figure 3:**
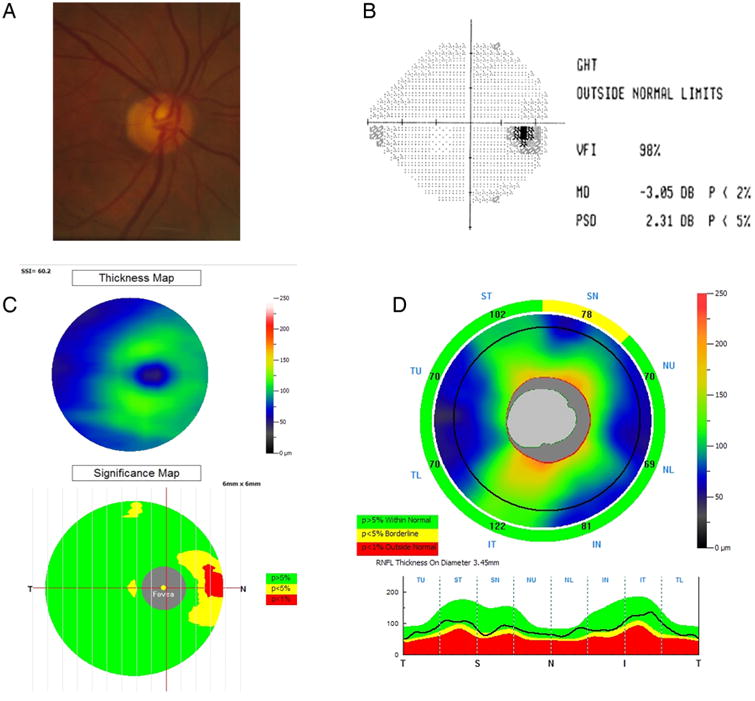
Example of the utility of the glaucoma structural diagnostic index (GSDI). Glaucoma was detected at 99% specificity cut-off using the combined GSDI (0.93) but not with any single optical coherence tomography (OCT) variable. (A) Disc photograph. (B) Visual field test (mean deviation (MD), pattern SD (PSD)). (C) Ganglion cell complex (GCC) thickness and significance map. (D) Retinal nerve fibre layer (RNFL) thickness profile.

Figure 3 illustrates a glaucomatous optic nerve and visual field with an abnormal GSDI at the p<0.01 level of a 63-year-old man. Glaucoma was detected at 99% specificity cut-off using the combined GSDI (0.93) but not with any single OCT variable, which were either normal (p>0.05) or borderline (p=0.05 to 0.01): the overall GCC (87.4 μm), GCC GLV (7.3%), NFL FLV (4.2%) and VCDR (0.76) were normal while GCC FLV (2.8%), overall NFL (81.0 μm) and NFL GLV (18.8%) were borderline. Conversely, there was no instance where GSDI missed the diagnosis and a single parameter was more accurate.

## Discussion

Recent advances in OCT imaging technology have resulted in instruments with greater resolution, shorter acquisition time and three-dimensional imaging of posterior segment structures. In the present study, we compared the discriminating power of a combined structural index generated from the peripapillary NFL, macular GCC and the optic disc to that of the individual parameters alone for glaucoma diagnosis.

The resulting GSDI was more sensitive in detecting glaucoma and outperformed single variables. This is consistent with a recent study that used a linear discriminant analysis to combine disc morphology, peripapillary NFL and inner macular thickness and demonstrated that diagnostic performance was improved over single variables.^13^ However, patients in that retrospective chart review had OCTexams already during the initial visit contributing to relatively high AUCs. A more accurate assessment of OCT performance may be achieved with independent, non-OCT diagnostic criteria as in the AIG Study participants analysed here, that are correlated well to traditional clinical parameters. Our results for single anatomical variables indicated a better performance of VCDR over horizontal cup to disc ratio as seen in established glaucoma prediction models that did not use OCT.^14^ The better performance of the inferior over the superior NFL is consistent with prior OCT studies.^15,16^

FD-OCT has been shown to have a good diagnostic accuracy for glaucoma detection with comparable power using GCC and NFL thickness.^17^ In the present study, global changes (GLV) had a better diagnostic performance when the anatomically larger GCC was weighted more than NFL, while focal changes (FLV) did better when NFL was weighted more heavily. Within the final multivariate logistic model for GSDI, composite overall thickness was weighed slightly more than focal loss changes and VCDR, but all three had an approximately even weight. Glaucoma was detected with considerably greater sensitivity than when single variables were used while false negatives were reduced and sensitivity increased at high specificity where it matters the most for diagnosis of a disease with a low prevalence: at 95% specificity, GSDI had a sensitivity of 80.8%, which was 12% better than NFL GLV. This means that with the use of GSDI to detect glaucoma, only 19 out of 100 patients with glaucoma would be missed, compared with 27 if the best single variable were used which represents a 44% reduction of false negative results. More importantly, the diagnostic performance in glaucoma with more serious visual field stages above GSS2,^10^ consistent with visual fields worse than, for example, a nasal step, was 100% at 95% specificity.

The diagnostic accuracy of GSDI is likely to be lower when applied to other study populations or clinic populations, which generally would not have exactly the same characteristics as the PG group in the AIG Study. However, we believe that the GSDI model captures patterns of anatomical change that is likely to be common in any open angle glaucoma populations. Specifically, either focal or diffuse loss of the NFL or GCC is likely to occur to similar degrees, and these are likely to occur in parallel with increase in vertical cupping (loss of disc rim in the superior and inferior poles). If other study or clinic populations share these characteristics, then GSDI is likely to have some benefit in increasing diagnostic accuracy over individual diagnostic variables.

The sensitivities we computed at 95% and 99% specificity cut-offs may help guide clinicians how to use this new variable. In addition to table 5 presented here, a GSDI formula spreadsheet can be accessed online.^18^ We suggest GSDI be software implemented by manufacturers directly on the device.

Since the first commercial software to map the macular GCC was introduced in an FD-OCT system (RTVue system by Optovue, Fremont, California, USA),^8^ similar software to map macular structures have become available on other platforms, for example, the Cirrus spectral domain optical coherence tomography (SD-OCT) (Carl Zeiss Meditec, Jean Germany), the Spectralis (HRA+OCT, Heidelberg Engineering, Heidelberg, Germany) and the RS-3000 (Nidek, Fremont, California, USA). The approach of combining diagnostic variables from three anatomical regions could therefore be implemented on several commonly used OCT systems. A recent study of SD-OCT similarly concluded that combination of diagnostic findings increases sensitivity by applying an OR-logic to minimal ganglion cell inner-plexiform layer and average NFL or minimal ganglion cell inner-plexiform layer and rim area.^19^

Despite our ‘leave-one-out’ cross-validation, a limitation of this study is that GSDI could only be tested on the same data set. GSDI resulted in improved diagnostic accuracy making it more useful in clinical situations where the pretest probability is increased when glaucoma risk factors are present. GSDI needs to be validated in larger studies, including population-based screening surveys. Using a single instrument, GSDI achieved a diagnostic performance that is similar to composite parameters that combine information from several instruments and that may have a sensitivity and specificity high enough to match screening algorithms that require multiple examination techniques.^20^ However, similar to prior studies comparing SD-OCT and time domain optical coherence tomography (TD-OCT) single variable analysis,^21–23^ we found that glaucoma diagnosis remains challenging at early stages and the performance of SD-OCT measurements was not notably better than TD-OCT when multiple anatomical areas are combined for glaucoma diagnosis.^24^

In conclusion, combining structural measurements of GCC, NFL and disc variables obtained with FD-OCT improved the diagnostic sensitivity for glaucoma compared with single variables while maintaining a high specificity.

## Supplementary Material

supplemental

supplemental table

## Figures and Tables

**Table 1:** Clinical and ocular characteristics of the study population

Clinical characteristics	Normal n=118	Perimetrical glaucoma n=236
Age (years)	58.5±9.1	60.0±8.6
Female	74 (63%)	150 (64%)
African-American origin	11 (10%)	29 (12%)
Axial length (mm)	23.8±1.0	24.4±1.4
Central corneal thickness (microns)	560±30	546±40
Intraocular pressure (mm Hg)	14.8±2.5	15.1±4.5
HVF mean deviation (dB)	−0.1±1.0	-5.0±4.5
HVF pattern SD (dB)	1.4±0.2	5.9±4.1
Visual field index (%)	99.5±0.6	87.5±13.5
Glaucoma stage		
Stage 0	64 (54%)	3 (1%)
Borderline	47 (40%)	3 (3%)
Stage 1	7 (6%)	90 (38%)
Stage 2	0	47 (20%)
Stage 3	0	45 (19%)
Stage 4	0	39 (17%)
Stage 5	0	9 (4%)

Enhanced Glaucoma Staging System (GSS2) used as detailed above.

HVF, Humphrey visual field.

**Table 2:** Relative weight of variables and diagnostic performance

Group	Variable	NFL weight (%)	GCC weight (%)	AUC after combining
1	Overall	63	37	0.894
2	Superior	72	28	0.857
3	Inferior	62	38	0.884
4	GLV	42	58	0.898
5	FLV	55	45	0.894

AUC, area under the receiver operator characteristic curve; FLV, focal loss volume; GCC, ganglion cell complex; GLV, global loss volume; NFL, nerve fibre layer.

**Table 3:** Multivariate logistic model for the glaucoma structural diagnostic index

Weighted variables	Weight (%)	OR	p Value*
Composite overall thickness (NFL+GCC)	38	2.52 per 10 μ thinner	<0.001
Composite FLV (NFL+GCC)	28	2.02 per 1% higher	<0.001
VCDR	34	1.40 per 0.1 higher	<0.001

* p Values were obtained from Wald's test.

FLV, focal loss volume; GCC, ganglion cell complex; NFL, nerve fibre layer; VCDR, vertical cup-to-disc ratio.

**Table 4:** Relevant high specificity cut-offs and sensitivities for best regional variables tested

Best regional variables	AUC	99% specificity cut-off	Sensitivity at 99% specificity	95% specificity cut-off	Sensitivity at 95% specificity (%)
GSDI	0.922*	0.80	69%	0.60	80.2
VCDR	0.867	0.85	51.5%	0.79	63.3
GCC FLV	0.844	3.9%	52.1%	2.3%	65.7
NFL FLV	0.884	8.3%	43.2%	5.1%	64.0
GCC GLV	0.886	11.6%	52.5%	9.3%	61.4
NFL GLV	0.896	23.1%	38.1%	14.5%	68.6
GCC overall	0.866	84.0 μ	50.0%	85.7 μ	56.4
NFL overall	0.894	76.5 μ	36.4%	86.1 μ	64.8

* The AUC, sensitivity and specificity for GSDI were calculated using leave-one-out cross-validation.

AUC, area under the receiver operator characteristic curve; FLV, focal loss volume; GCC, ganglion cell complex; GLV, global loss volume; GSDI, glaucoma structural diagnostic index; NFL, nerve fibre layer; VCDR, vertical cup-to- disc ratio.

**Table 5:** Diagnostic performance of GSDI at different glaucoma stages

Stage	# of cases	AUC*	Sensitivity at 99% specificity (%)	Sensitivity at 95% specificity (%)
<1	6	0.840	33.3	66.7
1	90	0.874	44.9	60.7
2	47	0.910	67.4	78.3
3	45	0.999	97.6	100.0
4–5	48	0.999	98.1	100.0

* The AUC, sensitivity and specificity for GSDI were calculated using leave-one-out cross-validation.

AUC, area under the receiver operator characteristic curve; GSDI, glaucoma structural diagnostic index.
