# Supplementary material for: Combining measurements from three anatomical areas for glaucoma diagnosis using Fourier-domain optical coherence tomography
Source: Br J Ophthalmol. Author manuscript; Available in PMC 2017 Jun 4. (PMC5457797; doi:10.1136/bjophthalmol-2014-305907)
Supplement: supplemental [file NIHMS845512-supplement-supplemental.pdf]

SUPPLEMENTARY MATERIAL

Data acquisition and processing

The RTVue software (version 6.12, RTVue system by Optovue, Inc, Fremont, CA, USA) was used to measure the overall, superior, and inferior hemisphere averages of (1) the GCC thickness map, (2) the NFL thickness profiles along the re-centered 3.4 mm circles, and (3) disc variables. Three scans of each type were performed on each visit and the measurements were averaged. Only scans with a signal strength index (SSI) parameter of ≥30 were saved. ONH scans with SSI>37 and GCC scans with SSI>42 were analyzed.

A pattern-based analysis was performed on the GCC thickness maps and NFL thickness profile data. The global loss volume (GLV) was used to measure a pattern of diffuse loss, whereas the focal loss volume (FLV) was used to measure localized loss. The loss volumes were normalized to the total GCC and NFL volumes in the measurement areas and presented as percentages. GCC, GLV and FLV were calculated by the RTVue software (6.12) based on Optovue's proprietary normal reference. We used our R group as the reference standard to define these new variables for NFL because FLV and GLV had not been defined in the RTVue software.

The OCT structural variables were combined into a single Glaucoma Structural Diagnostic Index (GSDI) in 2 stages in order to make optimal use of a large number of variables. First, we combined NFL and GCC thickness variables into 5 types that represented 5 patterns of possible glaucomatous loss: overall (avg), superior (sup), inferior (inf), GLV, and FLV. To obtain the proper weighting of the regions variables (avg, sup, inf) were first standardized (std) relative to their distributions in the normal reference group R, consisting of 105 eyes. This was done by subtracting the mean and dividing it by the standard deviation of the normal group: NFL_{avg_std} = (NFL_{avg} - 99.53)/8.04, NFL_{sup_std} = (NFL_{sup} -102.01)/8.94, NFL_{inf_std} = (NFL_{inf_std} - 97.04)/8.12, GCC_{avg_std} = (GCC_{avg} - 97.67)/7.17, GCC_{sup_std} = (GCC_{sup} -97.22)/7.12, GCC_{inf_std} = (GCC_{inf} - 98.12)/7.52. The GLV and FLV did not require standardization because they were already defined and calculated relative to the normal reference group. Logistic regression was used to optimize the weights of each standardized pair of NFL and GCC variables of the same type in the composite variables. P-values were determined with the Wald test. The weights provide insight into the relative importance of NFL and GCC for each type of variable. The five composite variables were then combined with the ONH variables into the GSDI by building a multiple logistic regression model with a stepwise selection procedure. The best diagnostic variables, ranked by AUC, were introduced one-by-one into the model ordered by significance

and were kept in the model if the significance level was less than 0.15. Diagnostic variables were eliminated from the model as new variable were introduced if their contribution was less significant than 0.15 using the likelihood ratio test. The final best multivariate logistic model produced the GSDI, which included the composite overall thickness, the composite FLV and the ONH VCDR. As a measure of model fit for the GSDI multivariate logistic model, the Hosmer-Lemeshow goodness-of-fit test was applied [23, 24]. The NFL GLV and FLV were calculated in the following manner. First, a pattern profile was determined by normalizing the NFL profile by dividing it by its own overall average. The fractional deviation profile and pattern deviation profile were then calculated. The fractional deviation profile was the NFL profile minus the normal reference profile, then divided by the overall average of normal reference. The pattern deviation profile was the pattern profile under consideration minus the normal reference pattern. The FLV was the sum of the fractional deviation profile in the region where there was significant focal loss. Significant focal loss was defined as both fractional deviation and pattern deviation more than 1.65 standard deviations below the fifth percentile of the normal distribution in R. The GLV was the sum of the fractional deviation in areas where it was negative. Diagnostic accuracy was assessed by determining the area under the receiver operating curve (AUC) to distinguish between PG and N eyes. The GSDI AUC was calculated using leave-one-out cross-validation at both stages of the 2-stage multiple logistic regression model building: RNFL and GCC of the same anatomic regions were combined while the normal group served as a reference for standardization. The coefficient of the multivariate model was then estimated. This method used a single observation from the original sample as the validation data, and the remaining observations as the training data to avoid the bias caused by testing on the training set itself. A generalized estimating equation (GEE) method was used for all analyses when applicable to adjust for potential correlation between two eyes from the same subject. All the statistical analyses were performed using SAS 9.2 (SAS Institute, Cary, NC, USA) software. The GENMOD procedure in SAS was used for the GEE. The resulting ROCs were correlated (same cohort) and clustered (up to two eyes per subject), then sensitivity and specificity for clustered data determined. We also calculated the partial AUC (pAUC) for GSDI from 90% - 100% specificity using the cross-validated curve and compared it to the best single parameter, NFL_GLV.
