# Supplementary material for: Combining measurements from three anatomical areas for glaucoma diagnosis using Fourier-domain optical coherence tomography
Source: Br J Ophthalmol. Author manuscript; Available in PMC 2017 Jun 4. (PMC5457797; doi:10.1136/bjophthalmol-2014-305907)
Supplement: supplemental table [file NIHMS845512-supplement-supplemental_table.pdf]

SUPPLEMENTARY MATERIAL

Supplementary Table 1: Anatomic Variables and Diagnostic Performance.

Variable Type	Normal	Perimetric Glaucoma	AUC
**NFL Variables**	Mean ± SD	Mean ± SD	
NFL Overall Thickness (microns)	99.5 ± 8.3	81.1 ± 12.5	0.894
NFL Superior Thickness (microns)	101.8 ± 8.7	84.2 ± 14.4	0.852
NFL Inferior Thickness (microns)	97.1 ± 8.9	78.1 ± 13.0	0.890
NFL Global Loss Volume %	6.27 ± 4.44	20.66 ± 10.42	0.896
NFL Focal Loss Volume %	1.59 ± 1.98	7.62 ± 4.86	0.884
**GCC Variables**			
GCC Overall Thickness (microns)	96.9 ± 6.5	83.6 ± 10.6	0.866
GCC Superior Thickness (microns)	96.3 ± 6.6	85.8 ± 11.1	0.794
GCC Inferior Thickness (microns)	97.4 ± 6.7	81.3 ± 13.0	0.864
GCC Global Loss Volume %	2.49 ± 2.62	13.28 ± 9.15	0.886
GCC Focal Loss Volume %	0.70 ± 0.82	5.17 ± 4.34	0.844
**Disc Variables**			
Rim Area (mm^2)	1.3 ± 0.3	0.8 ± 0.4	0.855
Rim Volume (mm^3)	0.16 ± 0.121	0.06 ± 0.06	0.861
ONH Volume (mm^3)	0.29 ± 0.17	0.12 ± 0.11	0.856
Cup to Disc Ratio: Vertical	0.55 ± 0.19	0.80 ± 0.16	0.867
Cup to Disc Ratio: Horizontal	0.65 ± 0.21	0.83 ± 0.17	0.773
Cup to Disc Ratio: Area	0.34 ± 0.18	0.60 ± 0.20	0.846

NFL = nerve fiber layer; GCC = ganglion cell complex; ONH = optic nerve head; AUC = receiver operator characteristic curve.
